# A stuck leaflet after balloon post-dilatation in transcatheter aortic valve implantation with a SAPIEN-3 ultra RESILIA valve: a case report

**DOI:** 10.1093/ehjcr/ytae697

**Published:** 2024-12-26

**Authors:** Shinji Yamazoe, Yasuhiro Ogawa, Naoaki Kano, Keita Mamiya, Katsuhiro Kawaguchi

**Affiliations:** Department of Cardiology, Komaki City Hospital, 1-20 Joubushi, Komaki, Aichi 485-8520, Japan; Department of Cardiology, Komaki City Hospital, 1-20 Joubushi, Komaki, Aichi 485-8520, Japan; Department of Cardiology, Komaki City Hospital, 1-20 Joubushi, Komaki, Aichi 485-8520, Japan; Department of Cardiology, Komaki City Hospital, 1-20 Joubushi, Komaki, Aichi 485-8520, Japan; Department of Cardiology, Komaki City Hospital, 1-20 Joubushi, Komaki, Aichi 485-8520, Japan

**Keywords:** Transcatheter aortic valve implantation, SAPIEN 3 Ultra RESILIA, Stuck leaflet, Transoesophageal echocardiography, Case Report

## Abstract

**Background:**

Transcatheter aortic valve implantation (TAVI) is a safe and effective therapy for patients with severe aortic stenosis. A Stuck leaflet and severe intraprosthetic regurgitation after valve implantation occur rarely but can lead to sudden haemodynamic deterioration. We encountered a case of a stuck leaflet following post-dilatation with the Edwards Sapien 3 Ultra RESILIA valve.

**Case summary:**

A 72-year-old woman was referred to our hospital for severe aortic stenosis with shortness of breath. She underwent transfemoral TAVI. After deployment of a 23 mm Sapien 3 Ultra RESILIA valve, post-dilatation was performed due to the presence of paravalvular leak (PVL). Transoesophageal echocardiography revealed a stuck leaflet and severe intraprosthetic regurgitation. Aortography also demonstrated severe aortic regurgitation. We performed valve-in-valve procedure using the second 23 mm valve. Post-valve-in-valve transoesophageal echocardiography showed no PVL nor aortic regurgitation, and haemodynamics improved.

**Discussion:**

A stuck leaflet is a rare complication following post-dilatation. Severe intraprosthetic regurgitation can lead to sudden haemodynamic changes and may, in some cases, necessitate the use of extracorporeal membrane oxygenation. If haemodynamic changes occur, it is essential to promptly investigate the cause through multiple diagnostic modalities, including transoesophageal echocardiography and angiography.

**Learning Points**

- Severe intraprosthetic regurgitation after valve implantation is rare but can lead to sudden haemodynamic deterioration.
- It is important to be knowledgeable about the management strategies for stuck leaflet.
- Transoesophageal echocardiography–guided monitoring allowed for the early detection and management of complications.

## Introduction

Transcatheter aortic valve implantation (TAVI) is a safe and effective therapy for patients with severe aortic stenosis. A stuck leaflet is a rare but severe complication of TAVI that can lead to acute haemodynamic deterioration.^1–5^ We report a case of a stuck leaflet occurring after post-dilatation with the Edwards Sapien 3 Ultra RESILIA valve.

## Summary figure

**Figure:**
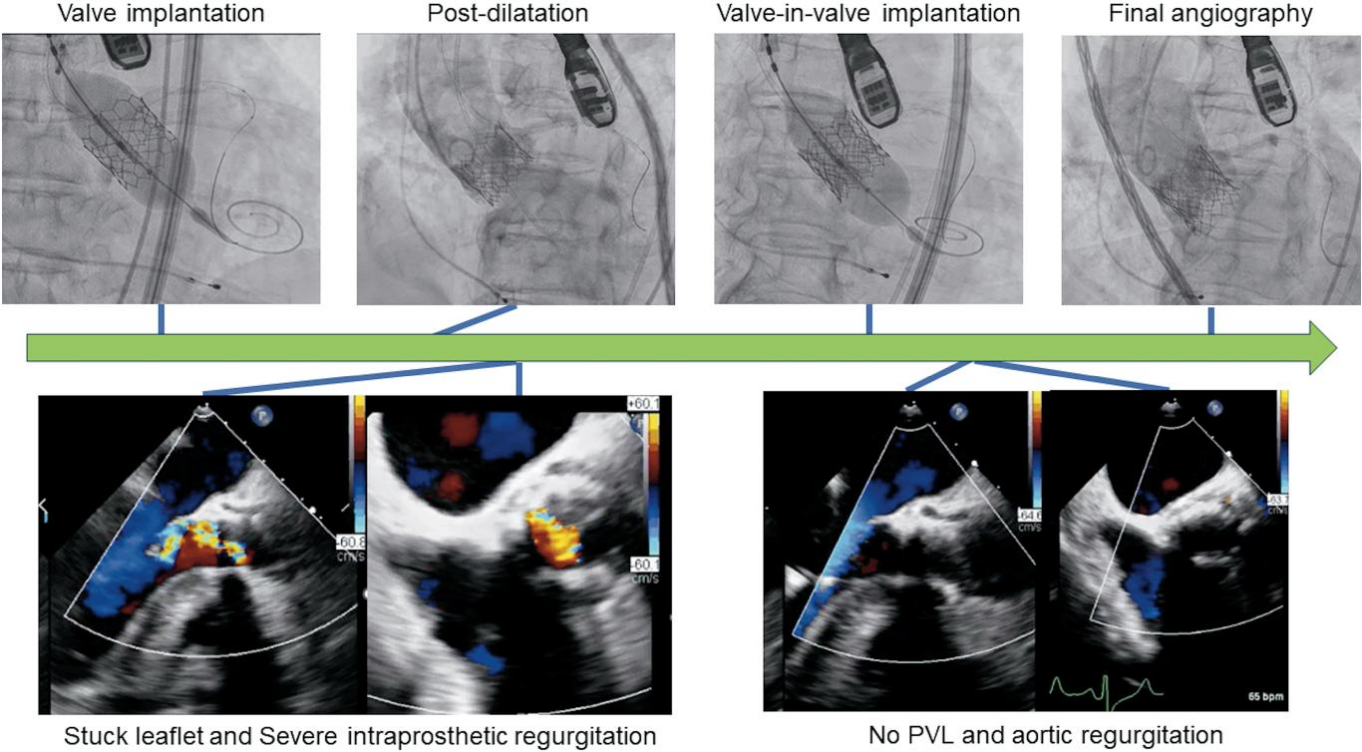


## Case presentation

**Figure 1:**
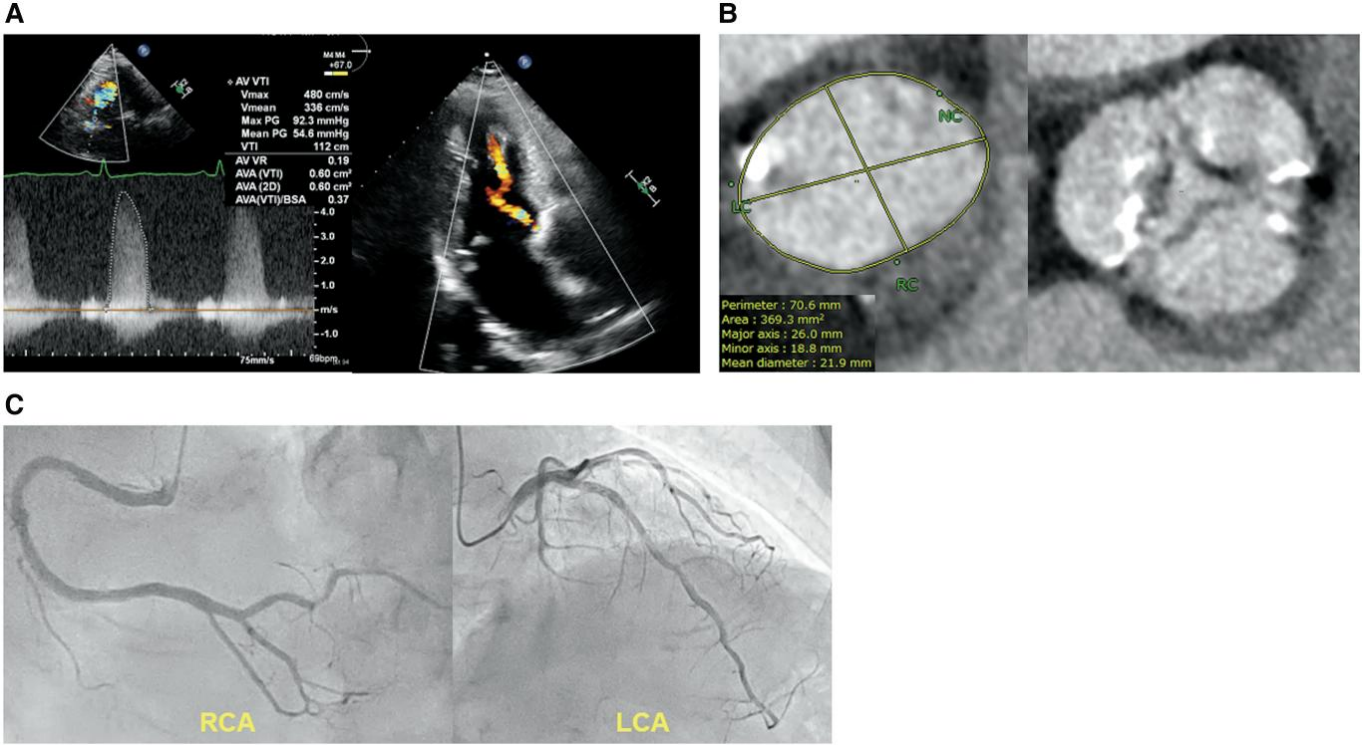
The preoperative imaging findings for the transcatheter aortic valve implantation procedure are presented. *(A)* Preoperative transthroracic echocardiography showed severe aortic stenosis and grade 1 aortic regurgitation. *(B)* Computed tomography revealed a tricuspid aortic valve with mild calcification. *(C)* Coronary angiography revealed no significant coronary artery stenosis.

**Figure 2:**
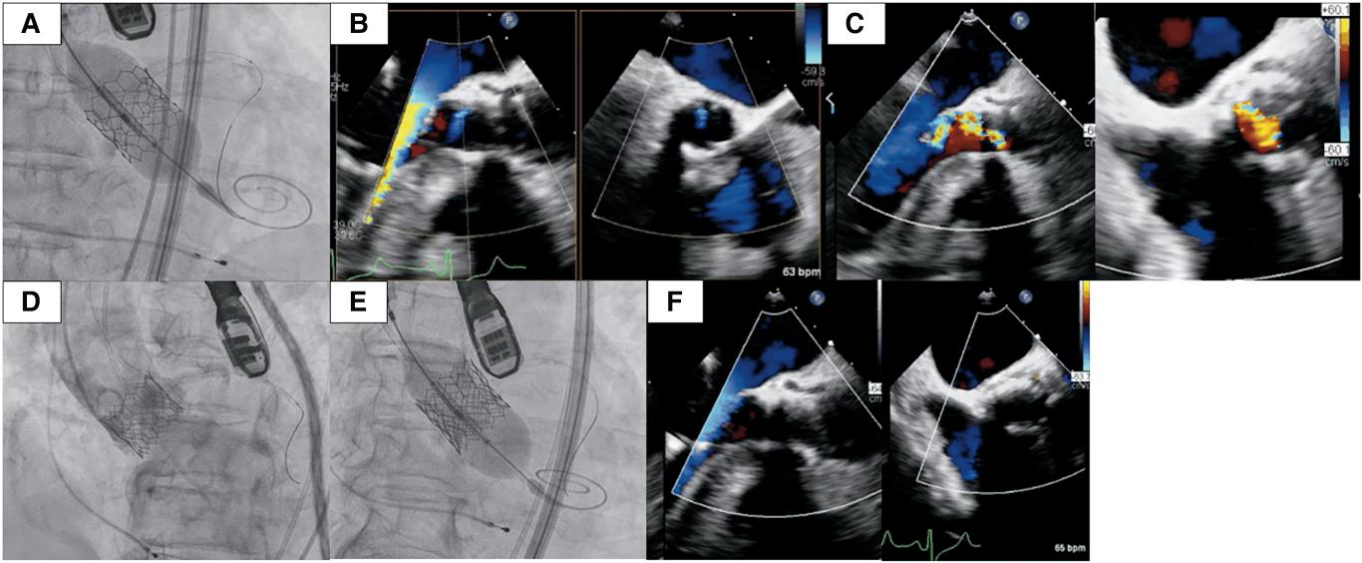
The operative findings during the transcatheter aortic valve implantation procedure are presented. *(A)* Sapien 3 Ultra RESILIA aortic valve with a diameter of 23 mm was deployed. *(B)* Transoesophageal echocardiography showed mild paravalvular leak following deployment. *(C)* Transoesophageal echocardiography demonstrated stuck leaflet and severe intraprosthetic regurgitation. *(D)* Angiography demonstrated Seller’s grade 3 aortic regurgitation. *(E)* Valve-in-valve procedure was performed. *(F)* Final transoesophageal echocardiography showed no paravalvular leak or aortic regurgitation.

**Figure 3:**
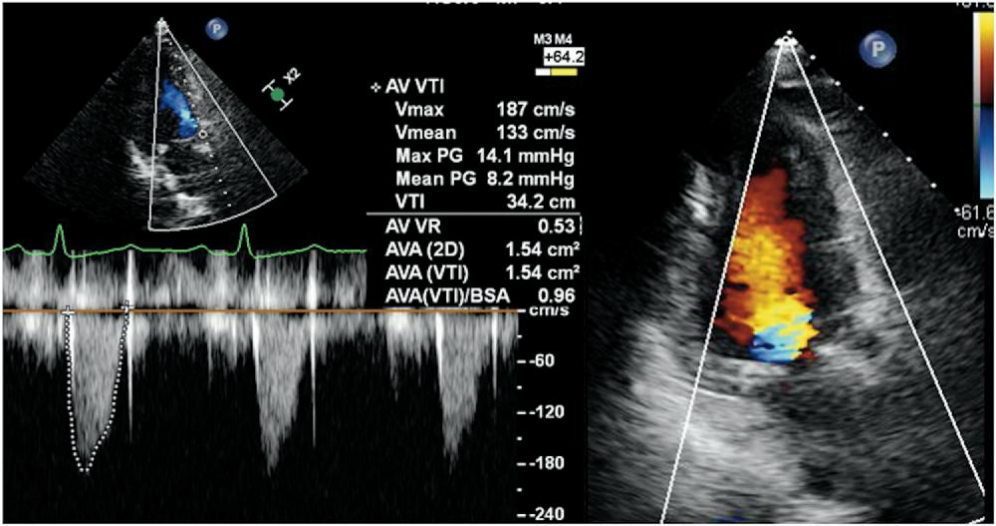
The post-operative transthoracic echocardiography demonstrated an improvement in pressure gradient, with no evidence of paravalvular leak.

A 72-year-old woman was referred to our hospital for severe aortic stenosis with shortness of breath. She had a history of diabetes and underwent surgery for a cerebral epidermoid cyst, during which an LP-shunt was placed. Additionally, she experienced recurrent nerve palsy following her brain tumor surgery. Her height was 153.5 cm, weight was 61.3 kg, and her BMI was 26.0. On examination, her blood pressure was 117/90 mmHg with a heart rate of 66 b.p.m. On cardiac auscultation, a systolic ejection murmur was present. Transthoracic echocardiography (TTE) showed a normal left ventricular ejection fraction, an aortic valve area of 0.6 cm^2^, peak velocity of 4.6 m/s, mean gradient of 55.7 mmHg, and grade 1 aortic regurgitation (AR; *Figure 1A*). Computed tomography showed an aortic annulus area of 369 mm^2^ and perimeter of 70.6 mm (*Figure 1B*). Coronary angiography revealed no significant coronary artery stenosis (*Figure 1C*). European System for Cardiac Operative Risk Evaluation [EuroSCORE II] score was 1.53%. After discussions within the heart team and considering the patient's decreased activities of daily living and the preferences expressed by both the patient and their family, the decision was made to proceed with TAVI. The procedure was planned through the transfemoral approach, under general anaesthesia (GA) and with transoesophageal echocardiography (TEE) monitoring. The peak-to-peak pressure gradient between the aorta and left ventricle, measured with a pigtail catheter, was 68 mmHg. The coronary height of the left coronary artery was 10 mm. After placing a wire in the left anterior descending artery for coronary protection, the Edwards Sapien 3 Ultra RESILIA aortic valve, with a diameter of 23 mm, was deployed under rapid pacing (*Figure 2A*). Mild paravalvular leak (PVL) was detected on TEE (*Figure 2B*), and post-dilatation was performed using a 23 mm balloon. Post-dilation was performed under rapid pacing; however, during the first attempt, the balloon position slightly shifted towards the aortic side, necessitating a total of two dilation procedures. Post-dilatation TEE revealed prosthetic valve leaflet motion and severe intraprosthetic regurgitation (*Figure 2C*, Supplementary material online, *Video S1*). Aortography confirmed Seller’s grade 3 aortic regurgitation (*Figure 2D*, Supplementary material online, *Video S2*). Additionally, the pigtail catheter could easily enter the left ventricle, and blood pressure decreased to 85/37 mmHg. We diagnosed a frozen leaflet following post-dilatation. We attempted to release the leaflet fixation using a pigtail catheter and a 0.035 inch guidewire, but the attempt was unsuccessful. Therefore, a valve-in-valve procedure was performed using the second 23 mm valve (*Figure 2E*). Post-valve-in-valve TEE showed no PVL or aortic regurgitation (*Figure 2F*, Supplementary material online, *Video S3*), and haemodynamics improved. The peak-to-peak pressure gradient between the aorta and left ventricle decreased to 7 mmHg, and no perioperative conduction disturbances associated with the TAVI were observed. Three days after the procedure, TTE demonstrated an effective orifice area of 1.6 cm², peak velocity of 1.9 m/s, and mean gradient of 7.5 mmHg, with no PVL detected (*Figure 3*). The patient was discharged on the tenth postoperative day.

## Discussion

Severe intraprosthetic regurgitation after valve implantation is rare but can lead to sudden haemodynamic deterioration. Instances of prosthetic valve stacking, termed ‘frozen leaflets,’ have been previously reported.^1–5^ Okuyama *et al*.^6^ reported a case of a ‘crashed valve’ following post-dilation. Prosthetic valve dysfunction during TAVI procedures has only been reported in case reports. It is primarily associated with balloon-expandable valves^1–4,6^ and often occurs after post-dilatation.^3,4,6^ However, Olasińska-Wiśniewska *et al*.^5^ have also reported instances involving self-expanding valves. Potential causes of a stuck valve include the following: (i) immobility of the prosthetic valve due to calcification of the native valve or aortic annulus, (ii) deformation of the valve resulting from post-dilatation, (iii) leaflet entrapment by the stent during crimping, and (iv) restricted valve movement due to asymmetrical stent-valve deployment.^3,7^ However, the precise mechanisms remain unclear. In cases of severe central regurgitation caused by a frozen cusp, the following measures may be considered: if haemodynamics are stable, adjust the position of the stiff guidewire, increase systolic blood pressure to assess if the frozen leaflet improves, attempt to release leaflet fixation using a pigtail catheter, and if the valve expansion is not circular, perform re-dilatation. If haemodynamics are compromised, extracorporeal membrane oxygenation should be considered as needed, and a valve-in-valve procedure should be performed.^7^ In this case, there were no abnormalities in leaflet motion or intraprosthetic regurgitation immediately after valve implantation. The regurgitation observed post-dilatation is thought to be due to deformation of the prosthetic valve resulting from the post-dilatation process. Attempts to release leaflet fixation using a pigtail catheter were unsuccessful, and with a subsequent decline in blood pressure, the decision was made to proceed with a valve-in-valve procedure. Transoesophageal echocardiography is a semi-invasive procedure that can lead to injuries of the oropharynx, oesophagus, and stomach, and it requires GA during TAVI. Recently, there has been an increasing shift towards performing TTE under monitored anesthesia care.^8^ On the other hand, De Agustin *et al*.^9^ have noted that the use of TEE during the procedure reduces the need for radiation exposure and contrast agents, facilitates the rapid detection of complications, and enables timely decision-making in complex situations requiring prompt intervention. In this case, TEE-guided monitoring allowed for the early detection and management of complications. The prognosis of PVL remains controversial. Some reports indicate that even mild PVL is associated with increased mortality and a higher rate of re-hospitalizations,^10,11^ while other studies suggest that only moderate or severe PVL is linked to an elevated mortality risk.^12,13^ While post-dilatation has been shown to reduce PVL,^14^ it can also lead to complications, as demonstrated in this case. Therefore, the decision to perform post-dilatation should be based on a comprehensive assessment of the degree of PVL and patient-specific factors. As a result, valve-in-valve implantation was necessary for this 72-year-old patient, raising concerns about potential issues with future reintervention. According to the Japanese Circulation Society guidelines,^15^ the patient’s preferences should be thoroughly considered when making the decision between surgical aortic valve replacement and TAVI. In this case, both the patient and their family strongly preferred TAVI, and therefore, the decision to proceed with TAVI is deemed appropriate. The patient initially had grade 1 AR, and the PVL after valve implantation was mild. In retrospect, it might have been preferable to accept the mild PVL and avoid further post-dilatation. A stuck leaflet is a rare but potentially severe complication, and it is important to be knowledgeable about the management strategies for this condition.

## Supplementary Material

ytae697_Supplementary_Data

## Data Availability

The data underlying this article are available in the article and in its online Supplementary material.

## Lead author biography

Shinji Yamazoe is a physician specializing in cardiology at Komaki City Hospital in Aichi Prefecture, Japan. After graduating from Ehime University School of Medicine and obtaining his medical license, he has dedicated his career to the field of cardiovascular medicine. His primary research interests include catheter interventions, structural heart disease, and echocardiography.

## Notes

**Consent:** The authors confirm that written consent for submission and publication of this case report including images and associated text has been obtained from the patients, according to COPE guidelines.

**Funding:** This research received no grant from any funding agency in the public, commercial or not-for-profit sectors.
